# Experimental Manipulation of Grassland Plant Diversity Induces Complex Shifts in Aboveground Arthropod Diversity

**DOI:** 10.1371/journal.pone.0148768

**Published:** 2016-02-09

**Authors:** Lionel R. Hertzog, Sebastian T. Meyer, Wolfgang W. Weisser, Anne Ebeling

**Affiliations:** 1 Department of Ecology and Ecosystem Management, Center for Food and Life Sciences Weihenstephan, Technische Universität München, Freising, Germany; 2 Institute of Ecology, University of Jena, Jena, Germany; Lakehead University, CANADA

## Abstract

Changes in producer diversity cause multiple changes in consumer communities through various mechanisms. However, past analyses investigating the relationship between plant diversity and arthropod consumers focused only on few aspects of arthropod diversity, e.g. species richness and abundance. Yet, shifts in understudied facets of arthropod diversity like relative abundances or species dominance may have strong effects on arthropod-mediated ecosystem functions. Here we analyze the relationship between plant species richness and arthropod diversity using four complementary diversity indices, namely: abundance, species richness, evenness (equitability of the abundance distribution) and dominance (relative abundance of the dominant species). Along an experimental gradient of plant species richness (1, 2, 4, 8, 16 and 60 plant species), we sampled herbivorous and carnivorous arthropods using pitfall traps and suction sampling during a whole vegetation period. We tested whether plant species richness affects consumer diversity directly (i), or indirectly through increased productivity (ii). Further, we tested the impact of plant community composition on arthropod diversity by testing for the effects of plant functional groups (iii). Abundance and species richness of both herbivores and carnivores increased with increasing plant species richness, but the underlying mechanisms differed between the two trophic groups. While higher species richness in herbivores was caused by an increase in resource diversity, carnivore richness was driven by plant productivity. Evenness of herbivore communities did not change along the gradient in plant species richness, whereas evenness of carnivores declined. The abundance of dominant herbivore species showed no response to changes in plant species richness, but the dominant carnivores were more abundant in species-rich plant communities. The functional composition of plant communities had small impacts on herbivore communities, whereas carnivore communities were affected by forbs of small stature, grasses and legumes. Contrasting patterns in the abundance of dominant species imply different levels of resource specialization for dominant herbivores (narrow food spectrum) and carnivores (broad food spectrum). That in turn could heavily affect ecosystem functions mediated by herbivorous and carnivorous arthropods, such as herbivory or biological pest control.

## Introduction

Current and future biodiversity loss strongly affects the functioning of ecosystems [1]. Arthropod communities depend on plant communities for food and habitat provision and shifts in plant species richness, plant biomass or plant functional composition lead to strong changes in the diversity of arthropod communities [2, 3]. During the last decades, many different studies, conducted in the framework of several experimental platforms, focused on the relationship between plant (diversity, productivity and composition) and arthropod communities. In the 1990s, e.g. Siemann et al. [2] reported an increase in herbivore, predator and parasite richness but not abundance, with increasing plant species richness. From their results, they concluded that plant species richness might not be the most important predictor for arthropod species richness and that interactions between trophic groups may maintain and promote overall diversity. During the same time, Koricheva et al. [4] presented contrasting responses of different arthropod orders and trophic levels to changes in plant species richness and plant community composition. For some orders, they found a positive response to increasing plant species richness while others showed negative or no response. More recently, Haddad et al. [5] identified different mechanisms for herbivores and carnivores driving the positive relationship between consumer richness and plant species richness. Scherber et al. [6] found higher species richness and abundances of all consumer trophic groups at higher plant species richness, with effect sizes decreasing with increasing trophic distance from the plant level, being strongest for herbivores. Classifying arthropod taxa into functional groups, as done by Rzanny et al. [7], showed that plant species richness had much smaller effects on arthropod functional group composition than the presence of legumes or plant biomass. The inconsistency in the observed patterns point out the need for studies replicating and extending previous results to derive general conclusions about the relationship between plant and arthropod diversity at different trophic levels.

Studies investigating the relationship between plant and arthropod diversity have mainly focused on arthropod abundance and species richness as response variables. However, abundance and species richness are only two out of many possible measures of arthropod community diversity. Wilsey et al. [8] showed that at least one additional index should be used that takes relative shifts in abundance into account (e.g. evenness or a measure of the abundance of dominant species). Adding such abundance-weighted measures may allow improved predictions for changes in arthropod mediated ecosystem functions [9–11]. Here we study the effect of plant species richness and plant community composition on herbivorous and carnivorous arthropods in a long-term biodiversity experiment, using four different, but complementary diversity indices, i.e. abundance, species richness, evenness and relative abundance of dominant species, hereafter: dominance, taking shifts in relative abundances into account.

A number of hypotheses link plant species richness and arthropod diversity [12], focusing on e.g. how resource diversity or resource amount change with increasing plant diversity. The examples and hypotheses we discuss in the following are valid for both herbivores and carnivores, despite the difference in their relationship to plant communities (herbivores use plant communities as food resource, whereas for their predators it serves as habitat and food resource for their prey) [2, 13]. Arthropod species have a varying degree of specialization to their food resources and as the number of host or prey species increase, potentially more arthropod species may find their food resources in the local habitat, resulting in a positive relationship between arthropod species richness and plant species richness. In its original version, this so-called ‘niche partitioning’ or ‘resource specialization hypothesis’ [14] does not make any predictions about shifts in arthropod community evenness or abundance of dominant species with increasing plant species richness. If the dominant consumer species have a narrow food spectrum they should be negatively affect by increasing plant species richness, as the density of the respective food resource decreases at higher species richness (‘food plant dilution effect’) [15]. As a result the expected increase in consumer species richness together with the decline in dominance along the plant diversity gradient would result in an unchanged consumer evenness. On the other hand, if the dominant species has a broad food spectrum increasing the number of available niches will not affect them. A second hypothesis considers the amount of resources as the main driver for changes in arthropod communities. The ‘productivity hypothesis’ state that the number of arthropod individuals is controlled by the productivity of the system, and as productivity increases, more consumer individuals can survive in the system due to higher availability of food resources [16]. Higher numbers of individuals in more productive systems can, as a consequence, affect arthropod species richness in two principal ways: i) sampling more individuals lead to more species being sampled (i.e. species accumulation curves [17]), or ii) with increasing population sizes, more species are able to persist [18]. As for the ‘niche partitioning hypothesis’, the ‘productivity hypothesis’ in its original version does not make any predictions about how productivity affects evenness in arthropod communities or the abundance of dominant species (dominance). Following the productivity hypothesis, we would expect, that the relationship between consumer evenness/ dominance and productivity depends on the dominance of the species in the community. As a general increase in population size particular benefit species with previously low population sizes, i.e. rare species, that are now able to persist in the community, increasing plant productivity will result in the presence of more species with lower population sizes in the community (decrease in dominance and increase in evenness).. If the general increase in population size is due to the increase in population size of dominant species, the dominance is likely to increase and evenness is likely to decrease. Because plant productivity increases with increasing plant species richness [19], the ‘niche partitioning hypothesis’ as well as the ‘productivity hypothesis’ make similar predictions regarding changes in arthropod diversity along a gradient in plant species richness. If plant species richness directly affects arthropod diversity, the ‘niche partitioning hypothesis’ can be identified as driving mechanism for the plant-arthropod diversity relationship. In contrast, the ‘productivity hypothesis’ can be stated as underlying mechanism if plant species richness effects are mediated by plant productivity and arthropod abundance. Beside plant species richness and plant productivity, arthropods can also be affected by other plant characteristics such as plant structural complexity and the consequent changes it induces in abiotic and biotic conditions i.e. increasing soil moisture [20] or more interspecific interactions [21]. Increasing plant species richness results in higher structural complexity [22]. Thus, not only resource niches but also niches created through changes in plant structure will increase with increasing plant species richness. Other important predictors of variation in arthropod diversity are plant functional composition [4, 7], plant functional diversity [23] or plant phylogenetic diversity [24]. Plant species within a functional group will share some properties of tissue quality affecting herbivores species feeding on them. For example, nitrogen-fixing plant species (legumes) have high nitrogen concentrations in tissues providing higher quality resource to herbivores [25]. Forbs with their large flowers and variable leaf size will increase the structural complexity of the habitat providing hiding places to prey species and affecting the efficiency of different hunting strategies [26]. Despite inconsistent and conflicting results reported in previous studies (see above), we expect a strong effect of legumes on all different aspects of herbivore and carnivore diversity. Herbivores may profit from higher nitrogen availability and carnivores from higher structural complexity when legumes are present (due to higher aboveground biomass).

We investigated the direct and indirect effects of changes in plant species richness, plant productivity and plant functional composition on abundance, species richness, evenness and dominance of herbivorous and carnivorous arthropods. Specifically, we asked: 1) How do plant species richness and plant productivity influence the different aspects of arthropod diversity (abundance, species richness, evenness and dominance) in experimental grasslands? 2) Are there differences in the plant diversity-arthropod diversity relationship between herbivores and carnivores? 3) Can we explain additional variation in arthropod diversity by the presence of specific plant functional groups in the plant communities?

## Materials and Methods

### Ethic statement

Plant and arthropod sampling was conducted with the permission of the city council of Jena, Germany.

### The Jena experiment

The data were collected in the framework of the Jena Experiment, located in eastern Germany (Thuringia, 50° 55’ N, 11° 35’ E; 130m above sea level). The experiment was established in 2002 and manipulates native plant species richness (1, 2, 4, 8, 16, and 60 plant species) in 80 plots of 400m^2^ size. Each diversity level was replicated 16 times except for the monocultures (14 replicates), 16-species mixture (14 replicates) and 60-species mixture (4 replicates) [27]. Species composition was randomly selected out of a species pool of 60 plant species, belonging to the native Arrhenaterio alliance. Based on 15 functional traits, the 60 plant species were classified into four plant functional groups, namely: grasses, small herbs, tall herbs and legumes. Using the four plant functional groups, a gradient in plant functional group richness from 1 to 4 was created, that is as orthogonal as possible to the species richness gradient. Due to variation in soil structure, four blocks were established. In 2009, the plot size was reduced to 6 x 9 m. The field site is mown twice a year, as is the common practice for managed grassland in this region. In addition, the plots are manually weeded three times per year to maintain the sown plant composition in the plots. The sown species richness and realized species richness have been shown to be highly correlated [28], and we therefore use the sown species richness in our analysis.

### Plant biomass

Aboveground plant biomass was collected in 2010 at peak standing biomass in May and August. In each plot, the vegetation was clipped three cm above ground within two randomly selected frames of 20 x 50cm. The plant material was sorted to species level, dried at 70°C for 72h, and weighted. Values from the two replicates were averaged and multiplied by ten to extrapolate to 1 m^2^.

### Arthropod sampling

Arthropod sampling was done in 2010 using two methods: a) pitfall traps, to sample ground living arthropods and b) suction sampling to sample vegetation living arthropods. Two pitfall traps with a diameter of 4.5cm were installed on each plot between May and September and emptied every two weeks. Traps were filled with a solution of 3% formaldehyde. For the suction sampling, a modified vacuum cleaner (Kärcher A2500, Kärcher GmbH, Winnenden, Germany) was used to sample all arthropods within a cage of 0.75m x 0.75m. Three random subplots where chosen on each plot and sampled in June and July between 9am and 4pm under optimal weather conditions (sunny, dry vegetation, little or no wind). Samples were conserved in a 70% ethanol solution before identification. Individuals were sorted to order or suborder level before being sent to specialist for species-level identification. Here we use data from Coleoptera, Hemiptera, Orthoptera, Araneae, Opiliones, Myriapods and Isopods individuals that were identified to species-level. Based on extensive literature search the species where classified in separate trophic levels (herbivores and carnivores) based on the food spectrum during their adult stage [29]. For Coleoptera we used feeding information from their larval stage, as this is the main feeding stage.

### Data Analysis

All analysis were done in R v3.1 [30]. Abundance data from the different spatial and temporal replicates were aggregated per plot and arthropod species. For each species, we only kept records sampled from the most appropriate method depending on the stratum where the species live [29]. To account for the different sampling intensity between the two sampling techniques, records from the two sampling methods were separately standardized by separating plot-level abundance for each species per sampling techniques and dividing these values by the maximum value obtained with the respective technique for any single species in any plot. That is all values obtained by the same sampling technique were standardized relative to the same value (See details in S1 File). To test for the effect of standardization methods we ran a sensitivity analysis comparing the impact of different standardization methods on the results (See details in S1 File and Table B in S3 File and Figures B-D in S3 File). We then summed per plot the standardized abundance. In addition to that, we computed three indices of diversity: species richness, Shannon evenness (see Eq (1)) and dominance (see Eq (2)). All these indices were computed for each plot (n = 80), separately for herbivores and carnivores based on the standardized abundance data.

$$\begin{array}{cccc} H & = & \frac{-\sum_{i=1}^{S}p_{i}*log_{e}\left(p_{i}\right)}{log_{e}S} & \text{where}\;\text{S}\;\text{is}\;\text{the}\;\text{species}\;\text{richness}\\ & & & \;\text{and}\;\text{p}\;\text{the}\;\text{relative}\;\text{abundance}\;\text{of}\;\text{the}\;\text{species.} \end{array}$$ (1)

$$\begin{array}{cccc} D & = & \frac{N_{1}+N_{2}}{\sum_{i=1}^{S}N_{i}}*100 & \;\text{where}\;\text{N1}\;\text{and}\;\text{N2}\;\text{are}\;\text{the}\;\text{abundance}\\ & & & \;\text{of}\;\text{the}\;\text{two}\;\text{most}\;\text{abundant}\;\text{species.} \end{array}$$ (2)

We also derived coverage-based rarefaction and extrapolation of the species richness to remove the effect of varying sample coverage on species richness [31]. Briefly, coverage-based rarefaction and extrapolation is more appropriate than abundance-based rarefaction as the ratios of species richness between samples is kept constant when using a coverage-based rarefaction and extrapolation. We used the protocol defined in Chao et al. [32] to compute the base coverage value and derived species richness at base coverage using the iNEXT package [33]. We controlled for block effects in the different indices by subtracting the plot-level value to the observed mean value of their respective block. Sown plant species richness was log-transformed as we expect log-linear relation between our diversity indices and sown plant species richness. Consumer abundance was log10-transformed, and evenness was logit-transformed to meet the assumptions of the parametric linear models used. We fitted linear models to the relation between all measured diversity indices separately and the log of the sown plant species richness. In addition, we also report in the Supporting Information the relation between the diversity measures and plant biomass. We used structural equation modelling (SEM) implemented in the lavaan package [34] to model the direct and indirect effect of plant species richness on our response variables: abundance, species richness, Shannon evenness and dominance of herbivores and carnivores. As independent variables, we included, beside plant species richness, plant biomass and the presence of plant functional groups. We only modeled paths from the plant functional groups to the total plant biomass for legumes and tall herbs following the results in Marquard et al. [35] that showed that only these two functional groups had significant effects on community level plant biomass in the plots of the Jena Experiment. We set the variance-covariance values between plant species richness and plant functional groups to the observed values for all models, as they result from our experimental design and should not be estimated by the model. The significance of particular effects might differ between the linear models and the SEM, as the set of variables included in the two types of model differ the fitted coefficients and their associated standard error will vary. We use linear model to explore the relationships between plant species richness and its biomass and the arthropod diversity indices, without controlling for the correlation between plant species richness and plant biomass but also for the effect of individual plant functional groups. The SEM on the other hand allow us to derive a mechanistic understanding of the observed patterns revealed by the linear models. As the 60 plant species mixtures are replicated only four times, and as they all have the same plant species composition, we conducted a sensitivity analysis, removing the 60-species plots and comparing the results including or excluding the 60-species mixture. All data used in this publication are published in the project database (www.the-jena-experiment.de)

## Results

In total, we sampled 14.142 carnivorous individuals belonging to 221 species (105 Araneae, 5 Opiliones, 93 Coleoptera, 4 Hemiptera, 5 Chilopoda), and 28.475 herbivorous individuals from 212 species (130 Coleoptera, 85 Hemiptera, 6 Orthoptera). Summary statistics of the various diversity indices derived from the herbivore and carnivore community data are given in Table A in S2 File.

### Herbivores

**Fig 1:**
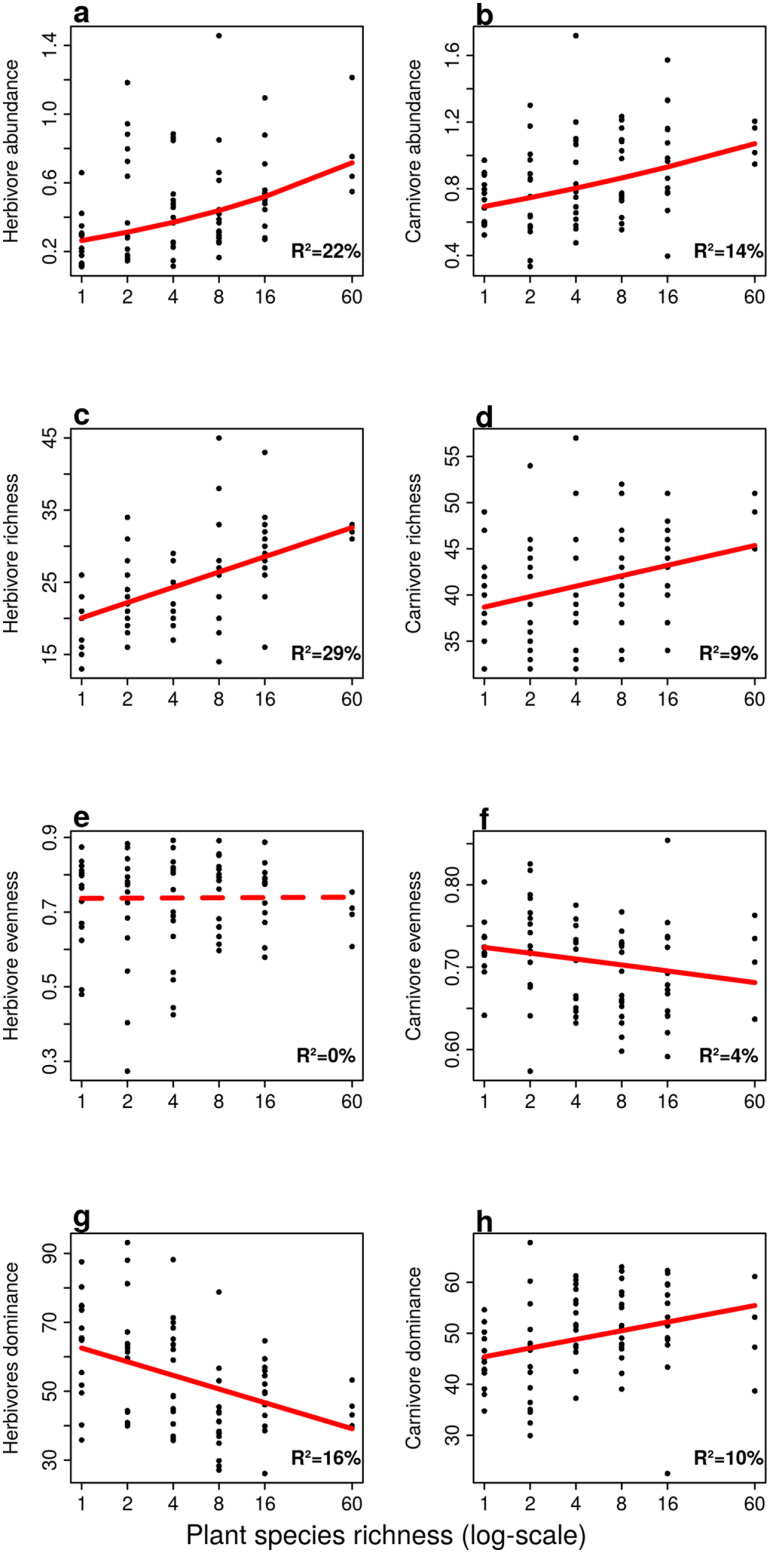
Bivariate relationships between plant species richness and the diversity indices. abundance (a and b), species richness (c and d), Shannon evenness (e and f) and dominance (g and h) for herbivores (left panel) and carnivores (right panel). All response variables were standardized by removing the block effect, ie by substracting from each experimental unit (n = 80) the average value measured in the respective block. Abundance was log10-transformed and evenness logit-transformed. The lines show fitted regression lines from linear models, and solid lines indicate significant diversity effects (p <0.05). The **R**^**2**^ values were taken from the linear models. Diversity indices and the fitted regression lines from the models were back-transformed to the original scale.

The linear models revealed a positive effect of plant species richness (PSR) on herbivore abundance, and species richness and a negative effect on the relative abundance of dominant species (dominance). Evenness in herbivore communities remained unaffected by changes in plant species richness (Fig 1).

**Fig 2:**
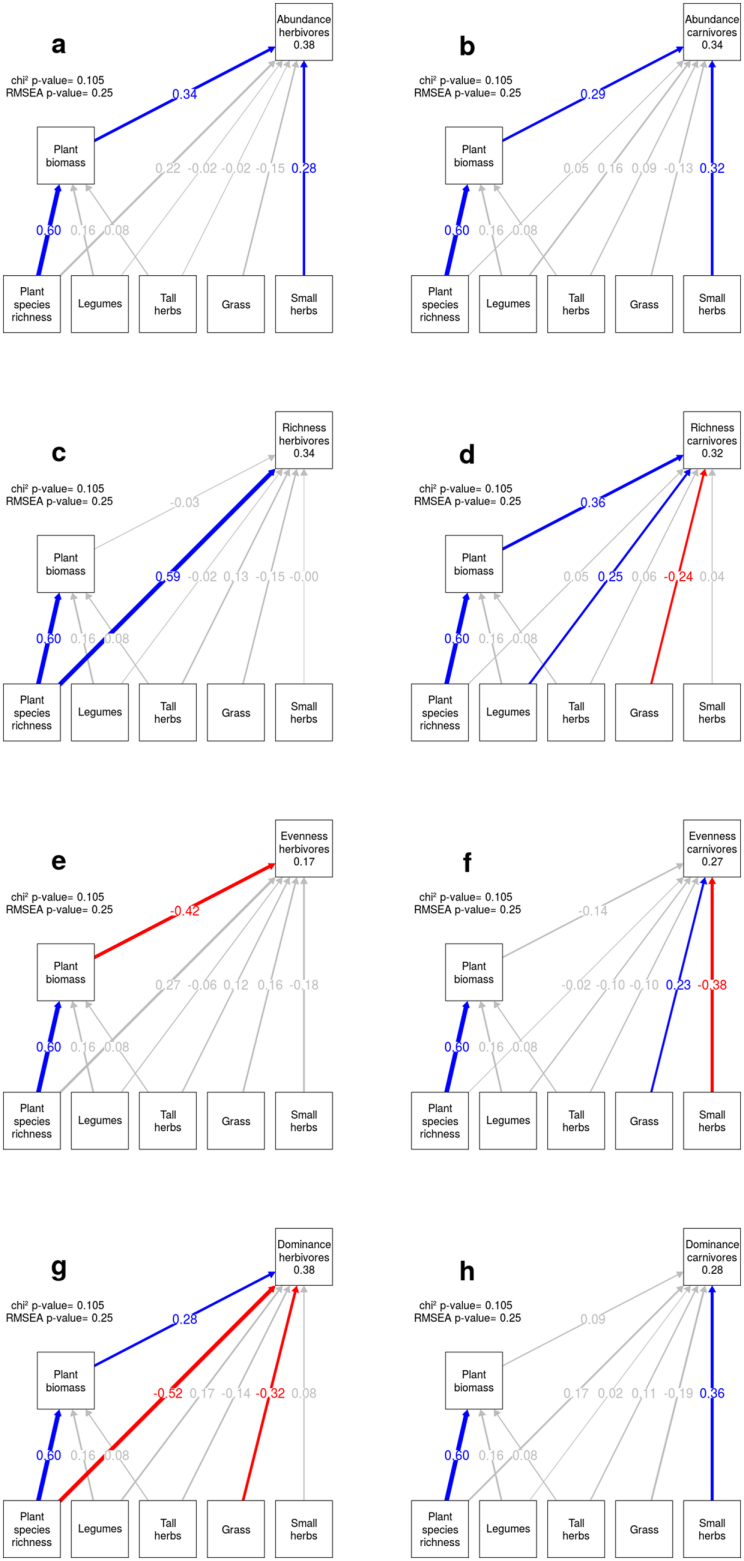
SEM’s representing plant community effects on arthropod diversity indices. Effects of plant sown richness, total plant biomass and the presence/absence of the four plant functional groups on abundance (a and b), species richness (c and d), Shannon evenness (e and f) and dominance (g and h) for herbivores (left panels) and carnivores (right panels). The reported path coefficients are standardized, and colored paths are significant at the 0.05 level. Blue paths have a positive path coefficient, whereas red paths have a negative one. We report the p-value for the Chi-square test of the SEM’s along with the p-value for the root mean square error (RMSEA). R^**2**^ value for the diversity indices are given in the indices box.

Using SEM’s, we could identify the mechanisms underlying the plant- herbivore diversity relationships (Table 1 and Fig 2). Effects of PSR on herbivore abundance were driven by changes in plant biomass, which increased with increasing PSR. Adding 100g/m^**2**^ of plant biomass led to an increase of 13% (95% CI: 3–20%) in herbivore abundance (see also Figure A in S2 File), and together with the positive non-significant direct effect of PSR on herbivore abundance, doubling PSR led to an increase in herbivore abundance by 19% (95% CI: 6–27%). Species richness of herbivores directly increased with increasing PSR. Doubling of PSR increased herbivore species richness by 2.4 species (95% CI: 1.1–3.4 species). This effect remained when herbivores species richness were rarefied (Figures B and C in S2 File). Doubling PSR led to a direct decrease in dominance by 5% (95% CI: -7, -2%), but on the other hand, increasing plant biomass by 100g/m^**2**^ increased herbivore dominance by 3% (95% CI: 0–5%, see also Figure A in S2 File). However, the negative direct PSR effect was stronger, leading to a total negative relationship between PSR and herbivore dominance. Herbivore evenness reflected the changes in herbivores dominance, as we identified a positive PSR (although non-significant) direct effect and a negative plant biomass effect on herbivore evenness (see also Figure A in S2 File). Direct and indirect PSR effect were of similar magnitude leading to a total effect close to zero. We observed only minor effects of single plant functional groups on the investigated diversity metrics. The presence of small herbs enhanced herbivore abundance, the presence of grasses and legumes lowered and increased herbivore dominance, respectively.

**Table 1:** Standardized SEM coefficients for herbivore diversity indices. Direct, indirect and total effects of plant species richness (PSR) on herbivore abundance, species richness, shannon evenness and dominance. Indirect effects are computed as the product of the PSR effect on plant biomass and the plant biomass effect on the diversity indices. Total effects are the sum of direct and indirect effects. Reported are also the effects of the four plant functional groups (Grasses, Legumes, Small Herbs and Tall Herbs). Coefficients in bold indicate a p-value <0.05.

Variables	Effects	PSR	Biomass	Grasses	Legumes	Small Herbs	Tall Herbs
Abundance	Direct	0.22	**0.34**	-0.15	-0.02	**0.28**	-0.02
	Indirect	**0.21**	-	-	0.05	-	0.03
	Total	**0.43**	-	-	0.03	-	0.01
Richness	Direct	**0.58**	-0.03	-0.15	-0.02	0.00	0.13
	Indirect	-0.02	-	-	-0.01	-	0.00
	Total	**0.56**	-	-	-0.03	-	0.13
Evenness	Direct	0.27	**-0.42**	0.16	-0.06	-0.18	0.12
	Indirect	**-0.26**	-	-	-0.07	-	-0.04
	Total	0.01	-	-	-0.13	-	0.08
Dominance	Direct	**-0.51**	**0.28**	**-0.32**	0.17	0.08	-0.14
	Indirect	**0.17**	-	-	0.04	-	0.02
	Total	**-0.33**	-	-	**0.21**	-	-0.12

### Carnivores

Linear models showed a positive effect of PSR on carnivore abundance, species richness and dominance (Fig 1), whereas evenness of carnivore communities declined along the gradient in PSR (Fig 1). The positive effect of PSR on carnivores species richness became non-significant when species richness was rarefied (Figures B and C in S2 File). SEM’s revealed that most of the PSR effect on carnivore diversity indices were either mediated by changes in plant biomass or the presence of particular plant functional groups (Table 2 and Fig 2). The positive effects of PSR on carnivore abundance and species richness were mediated by increases in plant biomass. Adding 100g/m^**2**^ of plant biomass led to an increase of carnivore abundance by 5% (95% CI: 0–10%, see also Figure A in S2 File) and added 1.0 more carnivore species (95% CI: 0.4–2.1 species) to the communities. The direct relationship between PSR and carnivores dominance was positive, but only became significant after removing the 60-species mixtures from the analyses. Doubling plant species richness increased the dominance in carnivore communities by 2% (95% CI: 0–3%), see also Table A and Figure A in S3 File. Shannon evenness of carnivores was unaffected by PSR, and plant biomass. The presence of small herbs increased the abundance of carnivores by 21% (95% CI: 7–36%), carnivore dominance by 6% (95% CI: 2–9%). As a result, carnivore evenness dropped by 0.19 (95% CI: -0.30, -0.08). The presence of grasses lowered carnivore species richness by 2.6 species (95% CI: -4.9, -0.3) but increased evenness by 0.12 (95% CI: 0.01–0.23). The presence of legumes enhanced carnivore abundance by 13% (95% CI: 0–28%), and increased carnivore richness by 3.4 species (95% CI: 1.1–5.7).

**Table 2:** Standardized SEM coefficients for carnivore diversity indices. Direct, indirect and total effects of plant species richness (PSR) on carnivore abundance, species richness, shannon evenness and dominance. Indirect effects are computed as the product of the PSR effect on plant biomass and the plant biomass effect on the diversity indices. Total effects are the sum of direct and indirect effects. Reported are also the effects of the four plant functional groups (Grasses, Legumes, Small Herbs and Tall Herbs). Coefficients in bold indicate a p-value <0.05.

Variables	Effects	PSR	Biomass	Grasses	Legumes	Small Herbs	Tall Herbs
Abundance	Direct	0.05	**0.29**	-0.13	0.16	**0.31**	0.09
	Indirect	**0.18**	-	-	0.05	-	0.02
	Total	0.22	-	-	**0.21**	-	0.11
Richness	Direct	0.05	**0.35**	**-0.23**	**0.24**	0.04	0.06
	Indirect	**0.22**	-	-	0.06	-	0.03
	Total	**0.27**	-	-	**0.30**	-	0.09
Evenness	Direct	-0.02	-0.14	**0.24**	-0.10	**-0.38**	-0.10
	Indirect	-0.09	-	-	-0.02	-	-0.01
	Total	-0.10	-	-	-0.12	-	-0.11
Dominance	Direct	0.17	0.09	-0.19	0.02	**0.36**	0.11
	Indirect	0.05	-	-	0.01	-	0.00
	Total	0.23	-	-	0.03	-	0.11

### Sensitivity analysis

Overall, there was no change in the observed patterns for herbivores after removing the 60-species mixtures, and only slight changes for carnivores. In detail, the total effect of PSR on carnivore species richness became non-significant, but the indirect effect of biomass remains. Further, the total effect of PSR on dominance becomes significant, due to an increasing direct effect of PSR (Table A in S3 File). Despite these qualitative and quantitative changes the direction of the effects remained positive. The second sensitivity analysis looking at the impact of the standardization method on the bivariate relationship as well as on the SEMs revealed that overall the results were consistent across different standardization methods. Only the negative effect of PSR on the abundance of dominant herbivores was not consistent when using different standardization methods (Table B and Figures B-D in S3 File).

## Discussion

Our investigation of common (abundance and species richness) and abundance-weighted (evenness and dominance) diversity indices revealed a strong influence of the plant community on herbivore and carnivore arthropod communities. Herbivore abundance and species richness increased with increasing plant species richness, dominance decreased and evenness was unaffected by PSR. SEM revealed that some of these effects were mediated by plant biomass (abundance, evenness, dominance), while others were directly linked to plant species richness (species richness, dominance). The functional composition of the plant community had little effects on herbivore diversity. Carnivores showed similar patterns of variation in common indices: abundance and species richness increased along the gradient in PSR. Further, dominance increased and evenness decreased with PSR. Decoupling between direct and indirect effects using SEM’s revealed that all PSR effects on carnivore diversity were mediated by plant biomass. The functional composition of the plant communities had a strong impact on carnivore diversity, especially the presence of small herbs (forbs of small stature), grasses and legumes.

### Sensitivity analysis

Removing the 60 species mixtures from the analyses indicates, that our results and observed mechanisms are robust, as there are no differences in the patterns of herbivores and for carnivores only in the total PSR effects (not direct and indirect effects). For carnivore species richness and dominance, the average values for the 60-species mixtures were outside of the 95% confidence intervals of the regression line fitted without these mixtures (Figure A in S3 File). This indicates that the observed carnivore richness and dominance in the 60-species mixtures is outside of what could be expected from all other mixtures (richness: higher than expected; dominance: lower than expected from mixtures). For dominance of carnivores, the lower than expected value could be an indicator for a quadratic relationship with PSR, and broadening the diversity gradient could reveal such a relationship. For carnivore species richness the higher than expected value might be due to accumulation of carnivores species in these mixture. A possible reason for both, higher and lower values can be found in the experimental design, as the 60-species mixtures are replicated only four times with the same plant species composition.

The comparison of different standardization methods revealed, that except for the bivariate relationship between PSR and the abundance of dominant herbivores, all patterns were robust (Table B in S3 File). In the following discussion we will assume that there is only a weak indication for the PSR effect on herbivore dominance and will thus not discuss this further.

### Plant species richness and plant productivity

The use of the common diversity indices, abundance and species richness, confirmed findings of many previous studies, which were conducted in experimental grasslands. For example, Haddad et al. [5] and Scherber et al. [6] showed higher species richness and abundance of carnivores and herbivores at higher plant species richness (except for herbivore abundance in [5] a negative relationship with PSR was observed). Similarly, Borer et al. [36] found increasing aggregated arthropod diversity with plant species richness. However, other studies [4, 7], separating arthropods into orders and/ or functional groups found PSR being a poor indicator of arthropod abundance compared to plant species composition. Moreover, these studies showed a high variability between single arthropod orders or functional groups in their response to PSR, likely caused by their different biology and relationships to plant communities. The inconsistency in the observed patterns between PSR and arthropod abundance and species richness, may arise from the differences between the study regions, the level of data aggregation or taxonomic resolution. However, there is consistency in the finding that aggregated arthropod species richness in grasslands increase with PSR irrespective of the trophic position [4–7, 36]. This general finding point out the importance of bottom-up effects in grasslands and the dramatic consequences of potential plant species loss on higher trophic levels. Using SEM allowed us to test for mechanisms, driving the effects of plant species richness on herbivore and carnivore communities. Our results for herbivore species richness support the ‘niche partitioning hypothesis’ as driving mechanism, as there was a direct effect of plant species richness. This robust pattern (still present after rarefying herbivore species richness) confirmed findings of other studies [5]. Our analysis using SEMs also revealed positive (herbivore abundance and dominance) and negative (herbivore evenness) effects of increasing biomass while controlling for PSR. With increasing biomass the total number of herbivores and the abundance of the dominant herbivore species increased, resulting in lower evenness of the herbivore community, and supporting the ‘productivity hypothesis’. Overall, we found for herbivores, that both mechanisms act in concert (‘niche partitioning hypothesis’ and ‘productivity hypothesis’), but ‘niche partitioning’ effects were stronger than ‘productivity’ effects. Our results for carnivore abundance and species richness support the ‘productivity hypothesis’, as higher values of both variables at high PSR, were driven by higher productivity. This pattern for carnivore abundance and species richness is in line with findings of others [2, 5]. We found that carnivore dominance increased with PSR, leading to the assumption, that higher general population sizes were driven by increases in dominant species. Higher dominance combined with higher species richness at higher PSR, led to a decline in carnivore evenness. However, adding the presence of plant functional groups to the models revealed that effects were mainly driven by changes in the plant community composition (see discussion below).

### Plant functional composition effects

Overall, we found strong effects of plant functional group composition on carnivores, but not on herbivore communities. Herbivore communities were little affected by plant functional group composition, what is in contrast, to what have been reported by Haddad et al. [5] or Rzanny et al. [7]. These authors revealed strong effects of legumes in structuring herbivore communities, which we could not confirm. There are several possible explanations for the differences in the observed legume effects and implications. 1) Differences in the observed legume effects might be caused by the fact that we explicitly separate legume effects from plant biomass effects in our analyses (but see [5]). 2) Second, there could be an increase in the top-down pressure of carnivores on herbivore abundance [37], which would mask the positive legume effect on herbivores. This option is supported by our data, as carnivore abundance increased when legumes were present. 3) Our results may also imply that in our studied grassland system the observed herbivores are not nitrogen-limited and are therefore not responding to the addition of nutrient-rich legumes [25]. Carnivore communities in our study were strongly affected by the plant community composition, as found by others [7]. Forbs of small stature increased the abundance of dominant species, leading to lower evenness in carnivore community. The presence of small forbs might influence carnivores by providing them with hiding places, easier anchorage point for casting webs or by harboring a specific herbivore fauna [38]. The presence of grasses led to higher evenness, but species-poorer carnivore communities, indicating, that the disappearing species are rare species. Indeed if the more abundant species were declining with the presence of grasses we would see this in a decline of the dominance index which is not found in our results (Fig 2). Moreover, species richness is mainly driven by species with low abundance so a decline in richness usually leads to a positive effect on evenness. Grasses have lower nutritious values than other plant functional groups and these lower values could cascade to the hosted herbivore community, making adaptations in the food spectrum of carnivores (e.g. increase in generalism) necessary [39]. Further, the presence of grasses could reduce the structural complexity of a habitat, which can have negative impact on carnivores through various mechanisms [21]. The presence of legumes increased the abundance of carnivores, maybe by providing more nutritious preys (herbivores) due to more nutritious plants [40].

### Functional consequences

The reported shifts in arthropod communities caused by changes in PSR and plant biomass may have consequences for ecosystem functions mediated by arthropods. Higher plant biomass at more diverse plant communities lead to more overall herbivore individuals, which has been shown to increase herbivory rates [41]. Further higher plant productivity leads to decreases in herbivore evenness and increases in the abundance of dominant herbivore species, indicating, that increases in productivity without concomitant increases in PSR (e.g. by fertilizing) may result in pest outbreaks. The above-predicted variation in herbivory rates, which could be caused by the presented shifts in herbivore diversity, will in turn affect other ecological processes such as plant growth [42], nutrient cycling [43] or plant community composition [44]. Higher plant biomass in more diverse systems increased carnivore abundance and dominance, and as we assume, that most of the carnivore species in our system are generalists we expect both to increase predation rates and hence also pest control. Our results indicate that effects on carnivore communities were mainly driven by plant productivity, which could be simply increased by fertilization, rather than by increasing PSR. But, we could further show, that the presence of single functional groups, which are only present if a high plant diversity is maintained in a system, enhance different aspects of carnivore communities.

### Conclusion

Our study confirms findings from other studies in experimental grasslands, reporting a positive relationship between PSR and common diversity measures (abundance and species richness) for herbivores and carnivores. Using abundance-weighted diversity indices (abundance of dominant species and community evenness) highlighted important differences between herbivores and carnivores. The abundance of dominant herbivores was unaffected by PSR while dominant carnivores showed a positive relationship with PSR, implying different levels of resources specialization for the dominant species of these two trophic levels. The reported shifts in arthropod diversity with PSR and the differences between herbivores and carnivores will have important consequences for the functioning of grassland ecosystem.

## Supporting Information

S1 File Information on the standardization of arthropod data. Here we give more detailed information on how we standardized arthropod data before analysing them, and on correlations between standardized and unstandardized data (Figure A).
(PDF)

S2 File Various supplementary informations. S2 File includes a summary statistic table (Table A), results of the relationship between arthropod diversity and plant biomass (Figure A), information on the effect of species richness rarefaction on bivariate models (Figure B) and on SEM’s (Figure C).
(PDF)

S3 File Sensitivity analyses. S3 File presents results of two sensitivity analyses. For the first, we removed the 60-species mixture from the SEM models (Table A) and from bivariate relations (Figure A). The second, tests for effects of different standarization methods on arthropod diversity (Table B and Figure B-D).
(PDF)
