# Supplementary material for: Experimental Manipulation of Grassland Plant Diversity Induces Complex Shifts in Aboveground Arthropod Diversity
Source: PLoS One. 2016 Feb 9;11(2):e0148768. doi: 10.1371/journal.pone.0148768 (PMC4747592; doi:10.1371/journal.pone.0148768)
Supplement: S1 File — Here we give more detailed information on how we standardized arthropod data before analysing them, and on correlations between standardized and unstandardized data (Figure A). (PDF) [file pone.0148768.s001.pdf]

Supporting Information for: Experimental manipulation of grassland plant diversity induces complex shifts in aboveground arthropod diversity

Lionel R. Hertzog, Sebastian T. Meyer, Wolfgang W. Weisser and Anne Ebeling

S1 File. Further explanations for the abundance standardization method used. To account for the variation of sampling intensity between pitfall traps and suction sampling we standardized the abundance for both methods separately. In a first step we determined for each species the best sampling technique based on information on the stratum in which the species lives. If both method could sample the species equally well we kept the records from the sampling technique giving the highest number of individuals. After this first step we had for each species, individuals sampled by only one sampling technique.
Then we divided the abundance values by the maximum value obtained with the respective technique for any single species in any plot (See equation 1). That is all values obtained by the same sampling technique were standardized relative to the same value. This maximum number gives us an indication of the sampling effort. For the suction sampling the maximum number of individual collected for one species was 381, for the pitfall traps it was 415. This brings every abundance value between 0 and 1, the unit is then the percent of individuals from the maximum value. A value of 0.2 meaning 20% of individuals from the maximum.

$$A_{ijk} = N_{ijk}/max(N_k) \tag{1}$$

For species i in plot j and sampled with method k.

In the third and final step we aggregated the data per plot and trophic levels summing up the individual species standardized abundance number (equation given below).

$$A_j = \sum_{i=1}^{N} A_{ij} \tag{2}$$

We then analyzed the variation in this standardized abundance. Going from 0.3 to 1 means that we increased the number of individuals from 30% to 100% of the maximum values. Below is a plot of the standardized abundance values against the original ones. The resulting standardized abundance values were highly correlated to the original ones (Fig. A)

To test for the effect of standardization methods on the patterns found in this study we conducted a sensitivity analysis using non-standardized data as well as two extra standardization method, which are suggested in the literature for this kind of data (Noy-Meir 1975):

$$A_{ijk} = N_{ijk}/sum(A_k) \tag{3}$$

$$A_{ijk} = N_{ijk}/mean(A_k) \tag{4}$$

Results from this sensitivity analysis are given in Supporting Information File 3.

Reference:

Noy-Meir, I., D. Walker, and W. T. Williams. 1975. Data transformations in ecological ordination. II. On the meaning of data standardization. Journal of Ecology 63: 779-800.

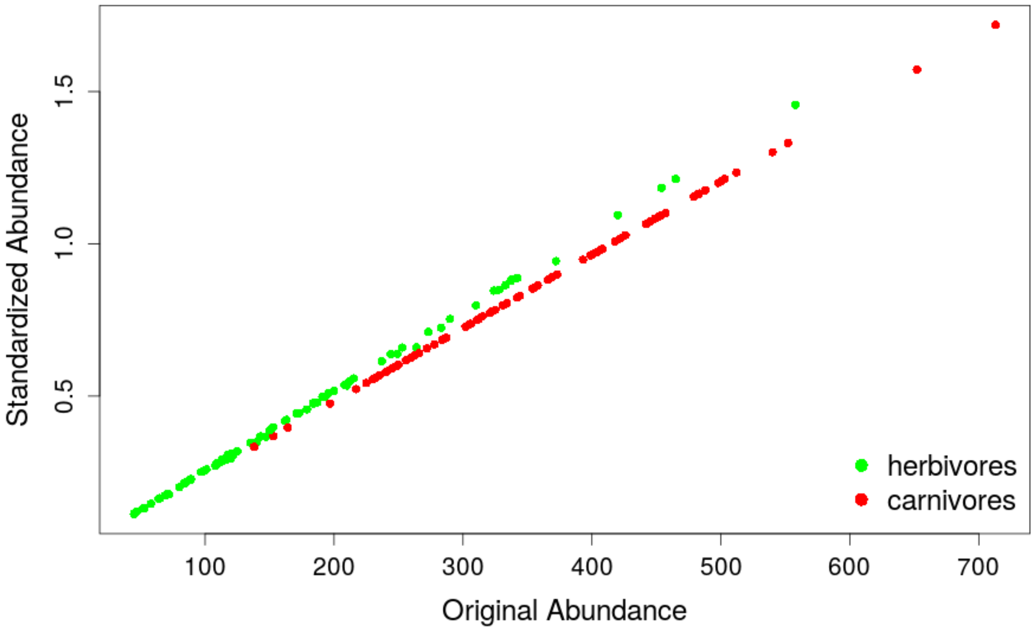

Figure A: **Standardized vs original abundance data at plot level for herbivores and carnivores.**
