# Supplementary material for: Experimental Manipulation of Grassland Plant Diversity Induces Complex Shifts in Aboveground Arthropod Diversity
Source: PLoS One. 2016 Feb 9;11(2):e0148768. doi: 10.1371/journal.pone.0148768 (PMC4747592; doi:10.1371/journal.pone.0148768)
Supplement: S2 File — includes a summary statistic table (Table A), results of the relationship between arthropod diversity and plant biomass (Figure A), information on the effect of species richness rarefaction on bivariate models (Figure B) and on SEM’s (Figure C). (PDF) [file pone.0148768.s002.pdf]

Supporting Information File 2 for: Experimental manipulation of grassland plant diversity induces complex shifts in aboveground arthropod diversity

Lionel R. Hertzog, Sebastian T. Meyer, Wolfgang W. Weisser and Anne Ebeling

Table A: **Summary statistics, n=80 plots**

	Abundance		Richness		Evenness		Dominance	
	Herbivores	Carnivores	Herbivores	Carnivores	Herbivores	Carnivores	Herbivores	Carnivores
Minimum	0.11	0.33	13	32	0.27	0.57	26	22
Mean	0.36	0.85	24	41	0.72	0.73	53	49
Maximum	1.46	1.72	45	57	0.89	0.85	93	68
Standard deviation	0.28	0.27	6.60	5.68	0.12	0.06	15.48	8.76

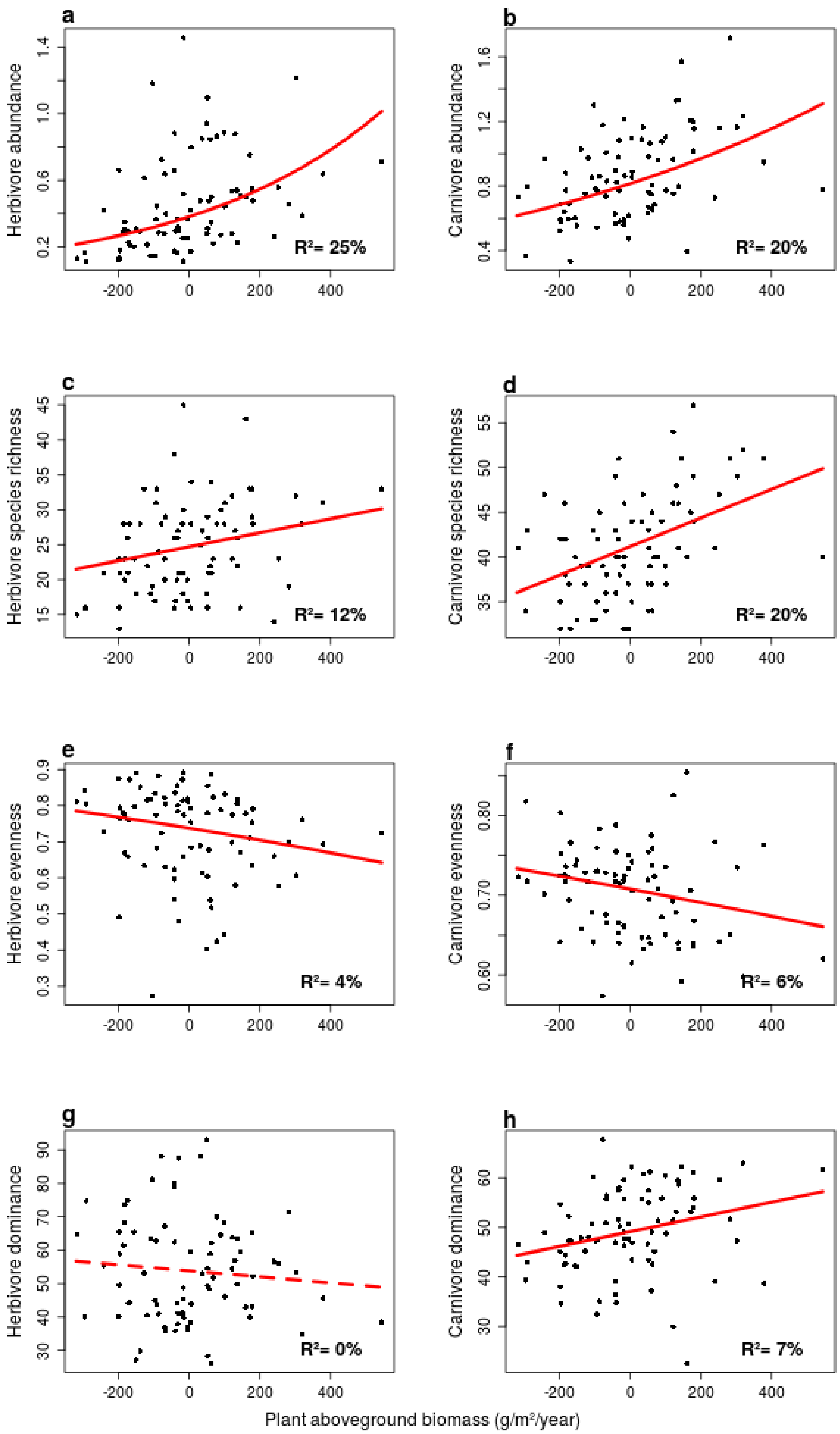

Figure A: **Relationship between the arthropod diversity indeces and plant biomass (g/m^2).** Abundance (a and b), species richness (c and d), Shannon evenness (e and f) and dominance (g and h). All response variables were standardized by removing the block effect,i.e. by substracting from each experimental unit (n=80) the average value in the block were the unit is. Abundance was log10-transformed and evenness logit-transformed. The lines show the fitted regression line, solid line indicate significance of the diversity effect ($p < 0.05$). The R^2 value is taken from the linear regression.

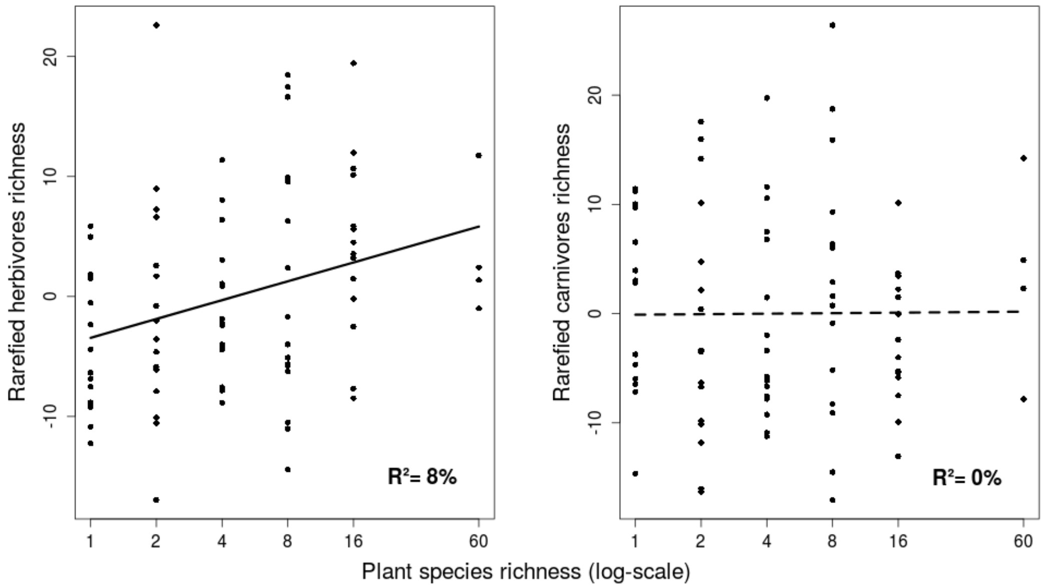

Figure B: **Relationship between rarefied species richness and plant species richness**, for herbivores (left panel) and carnivores (right panel). A thick line indicate a significant (p<0.05) slope. The R^2 value of the linear regression is given.

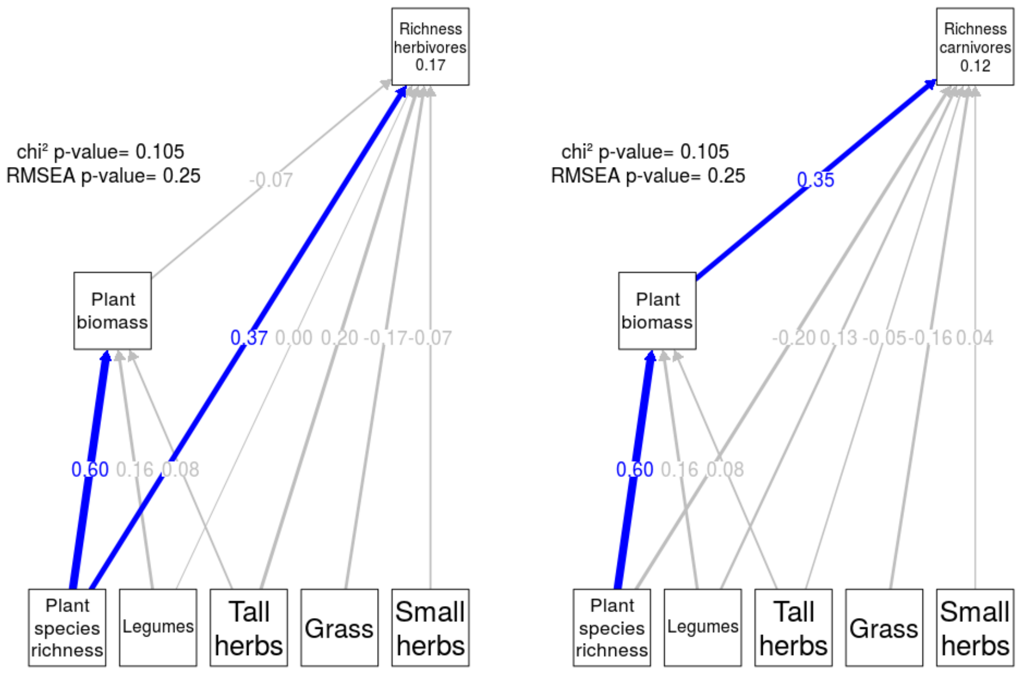

Figure C: **SEM models on the rarefied richness**, on the left side are the herbivores and on the right the carnivores, see the legend from Fig. 2 in the main text for further informations.
