# Supplementary material for: Experimental Manipulation of Grassland Plant Diversity Induces Complex Shifts in Aboveground Arthropod Diversity
Source: PLoS One. 2016 Feb 9;11(2):e0148768. doi: 10.1371/journal.pone.0148768 (PMC4747592; doi:10.1371/journal.pone.0148768)
Supplement: S3 File — presents results of two sensitivity analyses. For the first, we removed the 60-species mixture from the SEM models (Table A) and from bivariate relations (Figure A). The second, tests for effects of different standarization methods on arthropod diversity (Table B and Figure B-D). (PDF) [file pone.0148768.s003.pdf]

Supporting Information File 3 for: Experimental manipulation of grassland plant diversity induces complex shifts in aboveground arthropod diversity

Lionel R. Hertzog, Sebastian T. Meyer, Wolfgang W. Weisser and Anne Ebeling

Table A: **Sensitivity analysis of the effect of the 60-species mixture on the SEM results.**

a) Herbivores

Variables	Effects	PSR	Biomass	Grasses	Legumes	Small Herbs	Tall Herbs
Abundance	Direct	0.19	**0.31**	-0.15	-0.02	**0.28**	-0.02
	Indirect	**0.17**	-	-	0.05	-	0.03
	Total	**0.36**	-	-	0.03	-	0.01
Richness	Direct	**0.53**	-0.04	-0.15	-0.02	0.00	0.13
	Indirect	-0.02	-	-	-0.01	-	0.00
	Total	**0.51**	-	-	-0.03	-	0.13
Evenness	Direct	0.27	**-0.38**	0.16	-0.06	-0.18	0.12
	Indirect	**-0.21**	-	-	-0.07	-	-0.04
	Total	0.06	-	-	-0.13	-	0.08
Dominance	Direct	**-0.49**	**0.26**	**-0.32**	0.17	0.08	-0.14
	Indirect	**0.14**	-	-	0.04	-	0.02
	Total	**-0.35**	-	-	**0.21**	-	-0.12

b) Carnivores

Variables	Effects	PSR	Biomass	Grasses	Legumes	Small Herbs	Tall Herbs
Abundance	Direct	0.04	**0.29**	-0.13	0.16	**0.31**	0.09
	Indirect	**0.15**	-	-	0.05	-	0.02
	Total	0.19	-	-	**0.21**	-	0.11
Richness	Direct	-0.02	**0.34**	**-0.23**	**0.24**	0.04	0.06
	Indirect	**0.18**	-	-	0.06	-	0.03
	Total	0.16	-	-	**0.30**	-	0.09
Evenness	Direct	-0.09	-0.17	**0.24**	-0.10	**-0.38**	-0.10
	Indirect	-0.09	-	-	-0.02	-	-0.01
	Total	-0.18	-	-	-0.12	-	-0.11
Dominance	Direct	0.23	0.12	-0.19	0.02	**0.36**	0.11
	Indirect	0.07	-	-	0.01	-	0.00
	Total	**0.30**	-	-	0.03	-	0.11

Direct, indirect and total effect of the PSR, plant biomass (g/square m) and of the 4 functional groups on the abundance, richness and evenness of the a) Herbivores and b) Carnivores, without the 60-species plots. Reported are the standardized path coefficients from SEM, bold coefficient indicate significance.

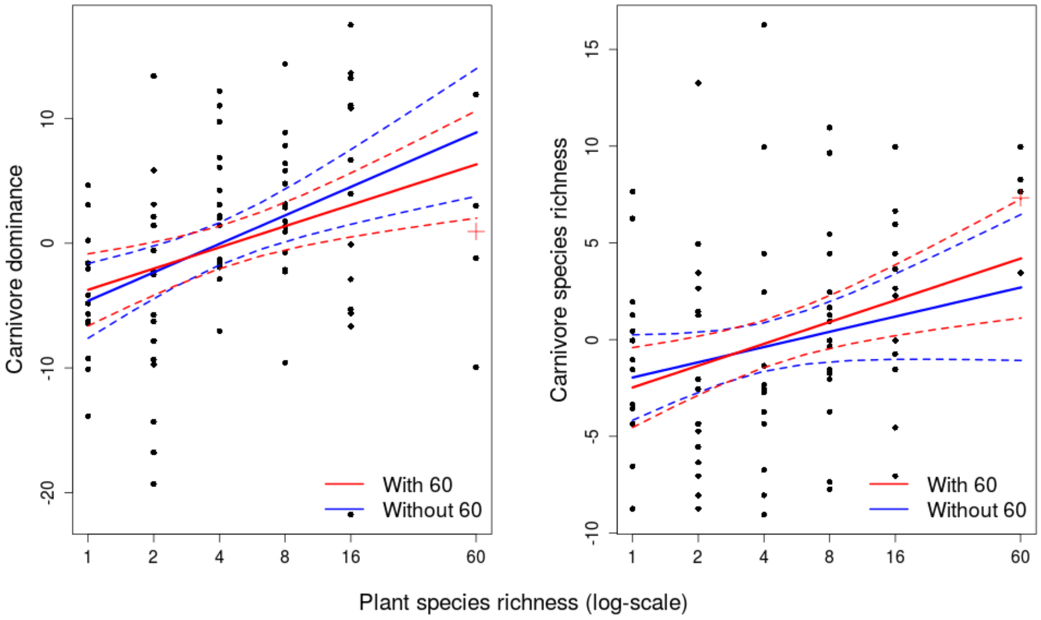

Figure A: **Relationship between plant species richness and carnivores richness (left) and dominance (right)**, the red line is the linear regression fitted line with the 60-species mixture while the blue line is without these mixture. The dotted line represent the 95% confidence interval around these fitted lines. the red cross is the mean observed value for the 60-species mixture.

Table B: **Sensitivity analysis of the standardization effect on the linear models**. Given are the slopes from the linear models together with their significance levels. Standardization 1 is the observed abundance divided by the maximum values of the respective sampling techniques (i.e. the method used in the main analysis). Standardization 2 is the observed abundance divided by the summed abundance of the respective sampling technique. Standardization 3 is the observed abundance divided by the mean of the respective sampling technique.

	Herbivores			Carnivores		
Standardization	Abundance	Evenness	Dominance	Abundance	Evenness	Dominance
Unstandardized	0.23^{***}	0.00	-2.22	0.11^{***}	-0.05^{*}	1.87^{*}
Standardized 1	0.24^{***}	0.00	-5.73^{***}	0.11^{***}	-0.05^{*}	2.46^{**}
Standardized 2	0.33^{***}	-0.13	0.57	0.11^{***}	-0.04	1.70^{*}
Standardized 3	0.27^{***}	0.00	-1.26	0.11^{***}	-0.05^{*}	1.84^{*}

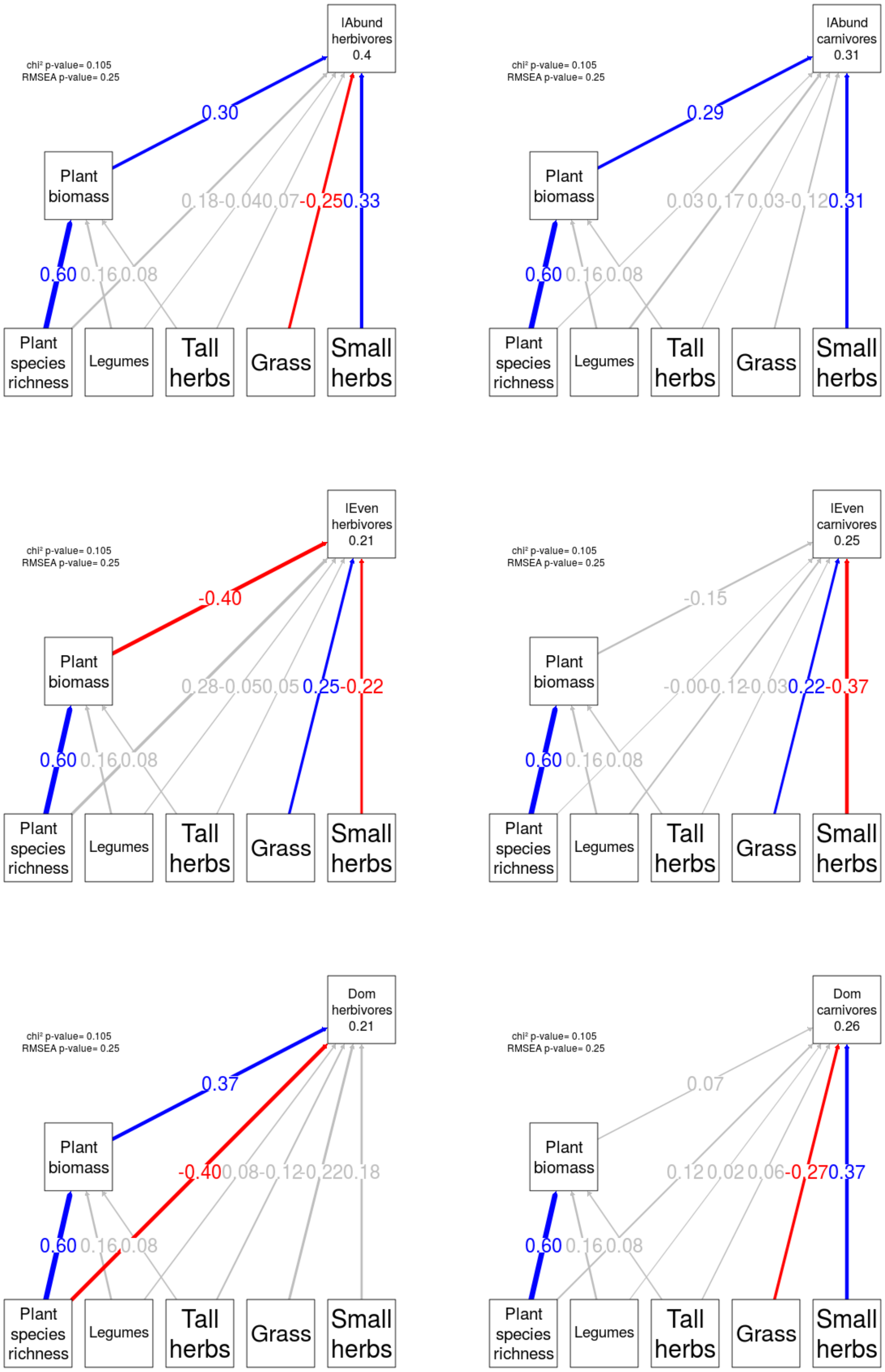

Figure B: **SEM models on the unstandardized data**, on the left side are the herbivores and on the right the carnivores, see the legend from Fig.2 in the main text for further informations.

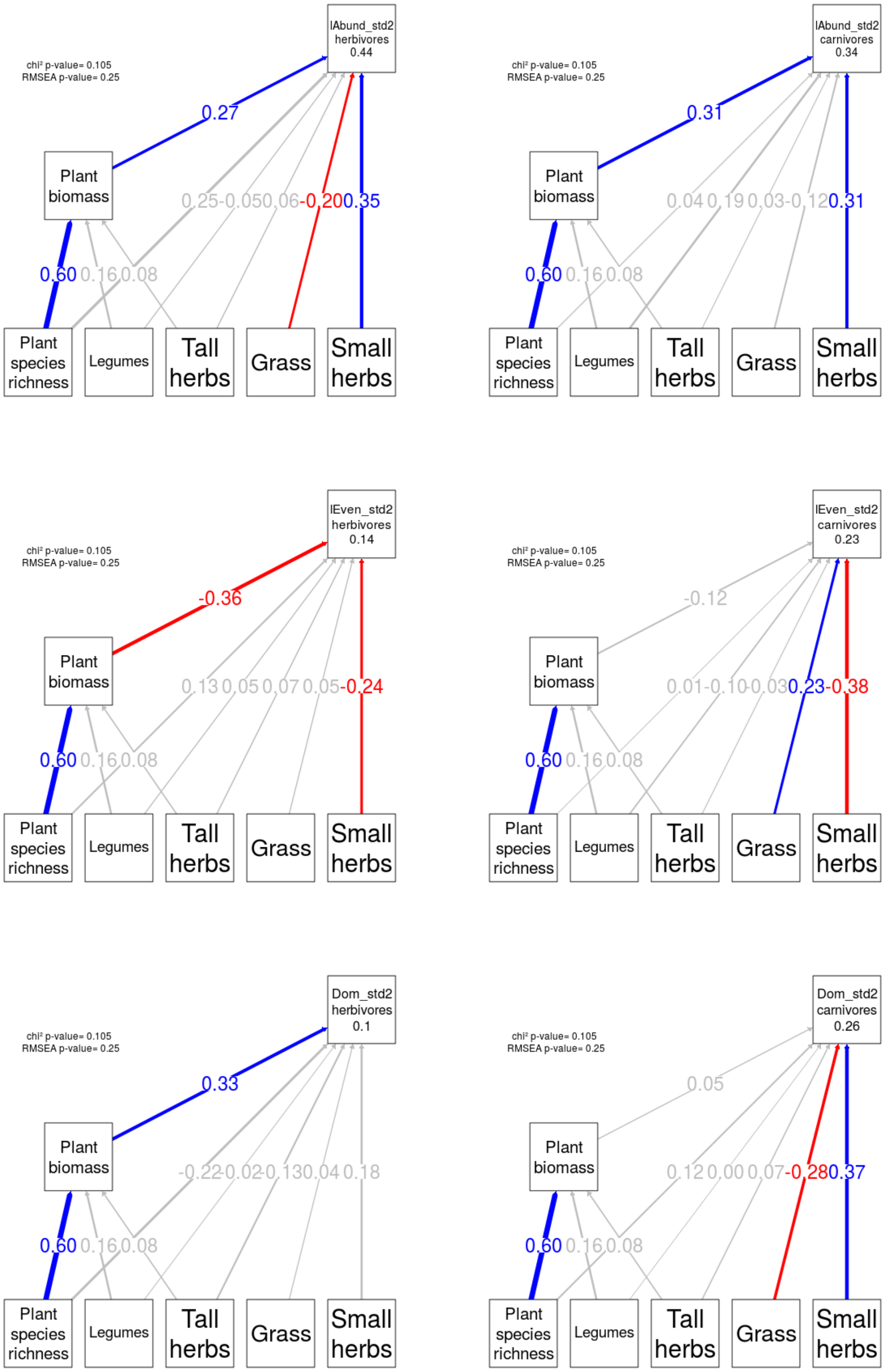

Figure C: **SEM models on the standardized by the sum data**, on the left side are the herbivores and on the right the carnivores, see the legend from Fig.2 in the main text for further informations.

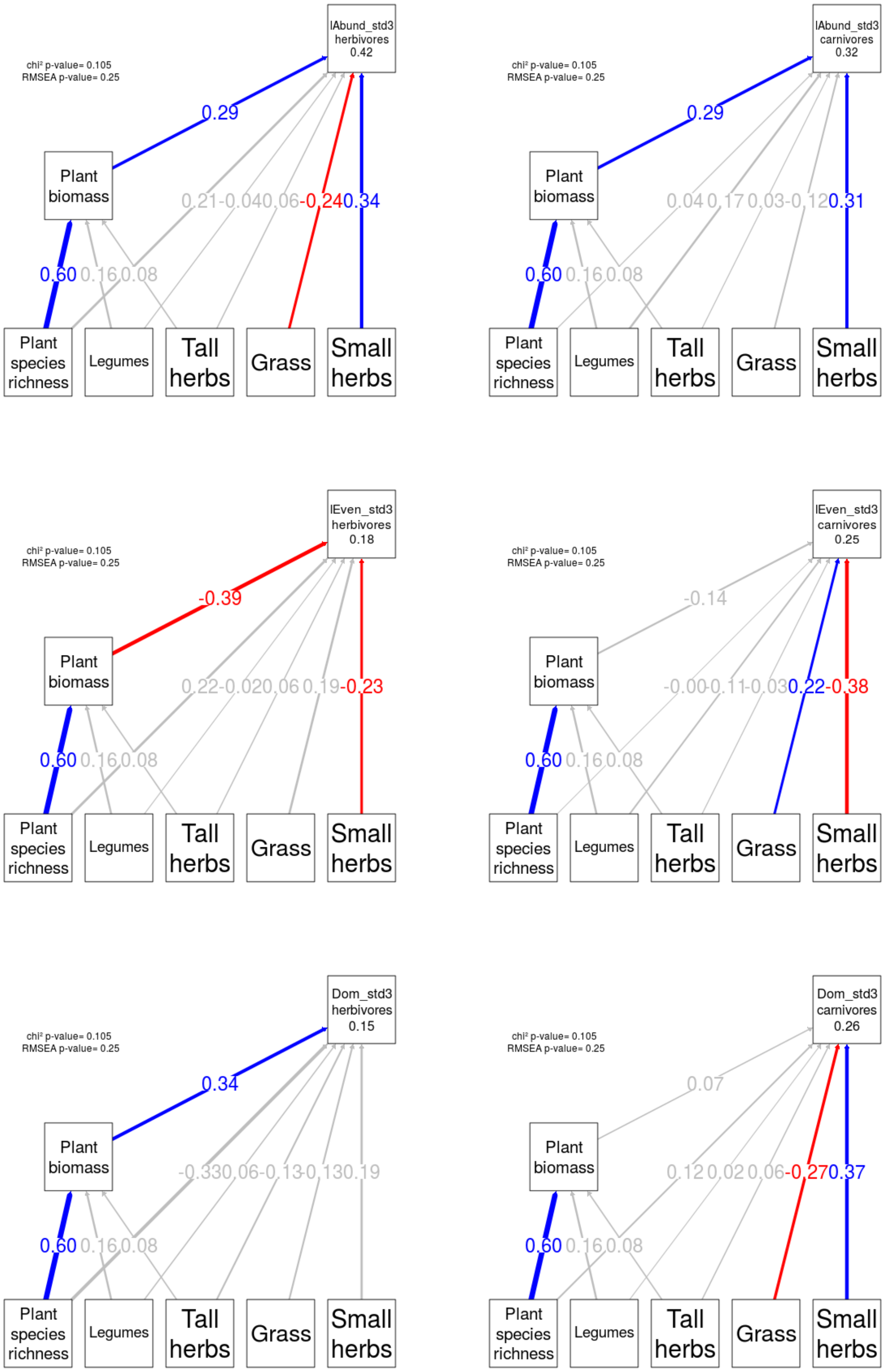

Figure D: **SEM models on the standardized by the mean data**, on the left side are the herbivores and on the right the carnivores, see the legend from Fig.2 in the main text for further informations.
